# A Journey through Time on the Discovery of Cell Cycle Regulation

**DOI:** 10.3390/cells11040704

**Published:** 2022-02-17

**Authors:** Rustem Uzbekov, Claude Prigent

**Affiliations:** 1 Faculté de Médecine, Université de Tours, 10, Boulevard Tonnellé, 37032 Tours, France; rustem.uzbekov@univ-tours.fr; 2 Faculty of Bioengineering and Bioinformatics, Moscow State University, Leninskye Gory 73, 119992 Moscow, Russia; 3 Centre de Recherche de Biologie Cellulaire de Montpellier, University of Montpellier, Centre Nationale de le Recherche Scientifique, CEDEX 05, 34293 Montpellier, France

**Keywords:** cell cycle, history, regulation, cyclin, cdk, checkpoint

## Abstract

All living organisms on Earth are made up of cells, which are the functional unit of life. Eukaryotic organisms can consist of a single cell (unicellular) or a group of either identical or different cells (multicellular). Biologists have always been fascinated by how a single cell, such as an egg, can give rise to an entire organism, such as the human body, composed of billions of cells, including hundreds of different cell types. This is made possible by cell division, whereby a single cell divides to form two cells. During a symmetric cell division, a mother cell produces two daughter cells, while an asymmetric cell division results in a mother and a daughter cell that have different fates (different morphologies, cellular compositions, replicative potentials, and/or capacities to differentiate). In biology, the cell cycle refers to the sequence of events that a cell must go through in order to divide. These events, which always occur in the same order, define the different stages of the cell cycle: G1, S, G2, and M. What is fascinating about the cell cycle is its universality, and the main reason for this is that the genetic information of the cell is encoded by exactly the same molecular entity with exactly the same structure: the DNA double helix. Since both daughter cells always inherit their genetic information from their parent cell, the underlying fundamentals of the cell cycle—DNA replication and chromosome segregation—are shared by all organisms. This review goes back in time to provide a historical summary of the main discoveries that led to the current understanding of how cells divide and how cell division is regulated to remain highly reproducible.

## 1. Principles

**Figure 1:**
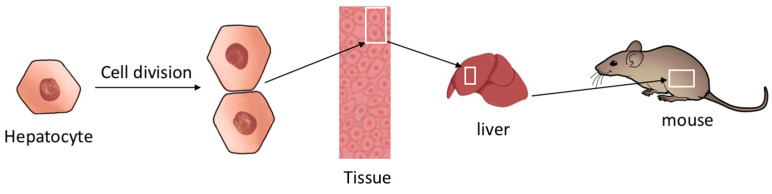
The cell is the smallest structural and functional biological unit of all living organisms. For example, the mouse liver comprises hepatocytes, which are the functional unit of the liver. As is the case for all cell types, all hepatocytes are generated by the division of another hepatocyte.

Described for the first time in plants in 1665 by Robert Hooke, the cell, from the Latin word *cellula* (monk’s chamber), ref. [1], is defined according to three main rules. The first rule is that “all living organisms are made of cells“, the second is that “the cell is the smallest structural and functional biological unit that constitutes a living organism”, and the third is that “every cell is derived from another cell” [2] (Figure 1).

How cells divide has been the subject of much research, initially most often performed using plant cells. In 1882, Walther Flemming, after developing methods to stain cells that revealed subcellular structures, was the first to draw different stages of the cell cycle leading to cell division [3].

**Figure 2:**
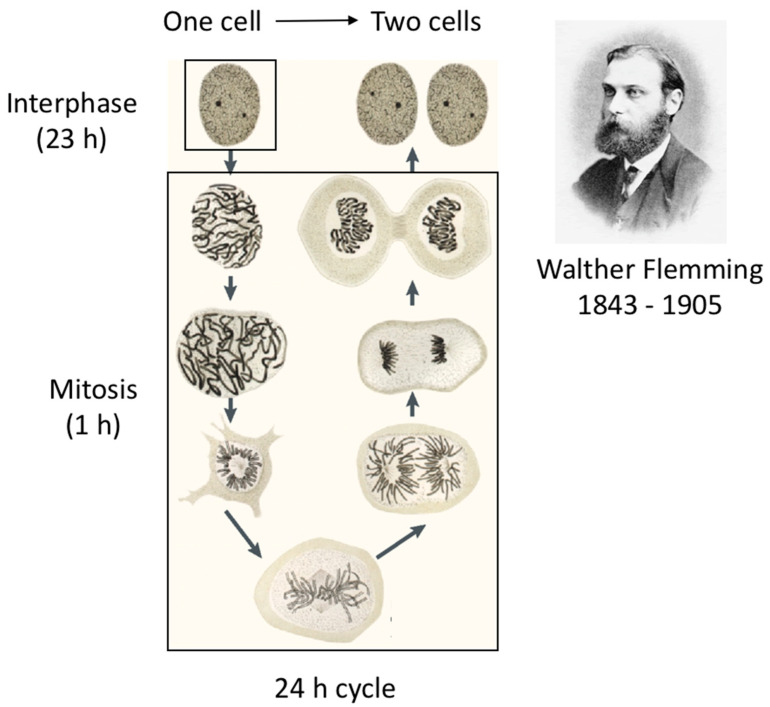
Drawing by Walther Flemming of a cell going through different stages of mitosis. (Image reproduced from ref. [3]).

For example, he documented and made drawings of a filamentous structure that changed in size and shape in the nucleus just before cell division takes place (Figure 2). Heinrich Wilhelm Gottfried von Waldeyer-Hartz baptised these filaments ‘chromosomes’, from the Greek words meaning colour (khroma) and body (soma) [4]. It was not known at the time that chromosomes contained the cell’s genetic information, although he knew that they were important cellular components. His drawings represent in astonishing detail the sequential events that occur over a short period of time before the physical separation of the two newly generated cells. However, during the longest part of the cell cycle, the cell appeared to be inactive and did not undergo any discernible changes, except that it increased in size (Figure 2).

When cells divide to give rise to two genetically identical cells, the process is referred to as the mitotic cell cycle, as opposed to meiosis, which is a specialised type of cell division corresponding to cells giving rise to one or four gametes. This name “mitotic” is a source of confusion, as researchers working on the mitotic cycle often describe the entire cell cycle as a mitotic progression. In contrast, researchers working on cell cycle progression consider mitotic progression as a progression through the mitotic phase of the cell cycle only, which is precisely the stage described in detail by Walther Flemming. Thus, the expression ‘mitotic progression’ should be restricted to the mitosis phase.

It was not until the mid-1900s that progress was made in regard to the understanding of the interphase. Alma Howard and Stephen R. Pelc, who had developed the autoradiographic technique [5], were the first to observe phosphorus-32 incorporation in the nucleus of meristem cells of the bean root *Vicia faba*, but only in cells that were dividing and, interestingly, only during a window of time in interphase [6]. Continuing the study of this incorporation of phosphorus-32, they proposed dividing the interphase of the cell cycle into three phases: the G1 phase (for Gap1), the S phase (for synthesis) corresponding to DNA synthesis (whereby ^32^P is incorporated into the DNA), and the G2 phase (for Gap2). The entire cell cycle was hence considered to comprise four stages: mitosis, G1, S, and G2 [7]. The same year, 1953, saw the pivotal discovery of the structure of DNA by Francis Crick and James Watson, which had extraordinary consequences regarding the understanding of the cell cycle and, in particular, the mechanisms of gene transmission from the original cell to its newly generated cells [8,9]. S phase, corresponding to DNA synthesis, is the stage during which the cell duplicates its genome to generate two copies, one copy for each newly generated cell. A year later, Laszlo Lajtha and co-workers reported that animal bone marrow cells also obey this four-stage cell cycle rule [10], which was a landmark finding suggesting the universality of the cell cycle. Laszlo Lajtha also made a great discovery by finding that cells can exit the cell cycle to enter a resting phase, called G0, during which cells can rest several years while waiting for a signal to re-enter the cell cycle and then divide or differentiate [11].

**Figure 3:**
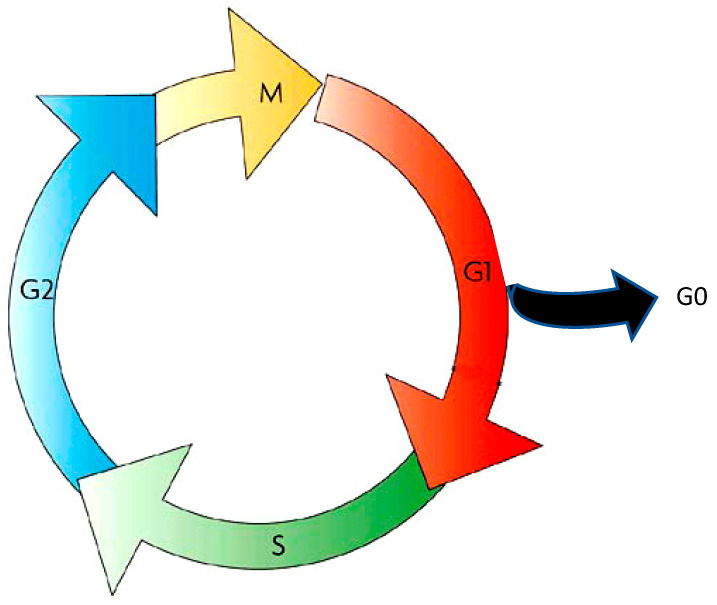
The different stages of the cell cycle, shared by all eukaryotic cells.

In the 1960s, the cell cycle was thus defined as a succession of four phases—G1, S, G2, and M—plus an extra G0 phase (Figure 3).

## 2. The First Cell Cycle Control Discovery: The Cell Cycle Engine

Several fields that were working separately eventually joined forces to elucidate what controls the cell cycle: zoologists working on frog development, biochemists who used frog eggs as a test tube, geneticists who studied yeasts, and, to a lesser extent, cell biologists.

**Figure 4:**
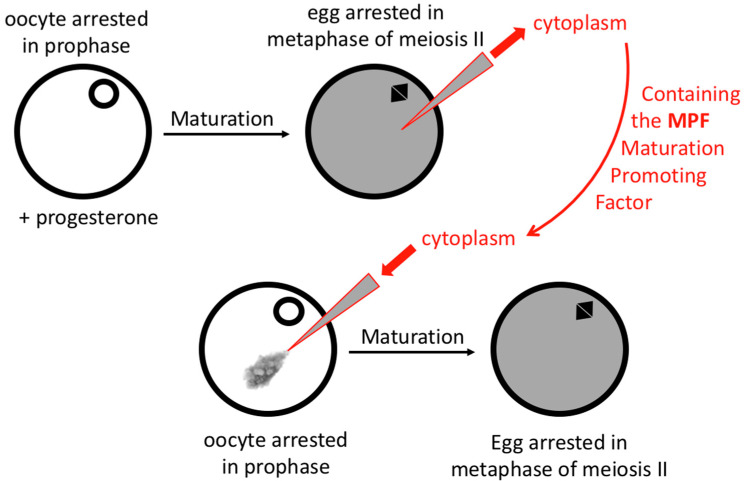
The discovery of MPF (Maturation Promoting Factor) by manipulation of the transition from frog oocytes arrested in prophase to eggs arrested in metaphase of meiosis II.

Starting with zoologists, Yoshio Masui made a giant step toward understanding cell cycle controls. He chose to work with frog oocytes because they arrest naturally at specific stages of the cell cycle: oocytes in prophase and eggs in metaphase of meiosis II. In 1967, Masui’s team showed that the addition of progesterone to isolated oocytes arrested in prophase was sufficient to trigger their maturation to eggs arrested in metaphase of meiosis II [12] (Figure 4). More importantly, in 1971, they also demonstrated that a small fraction of the cytoplasm of an egg injected in a prophase oocyte was sufficient to trigger the same transition, thus providing an essential assay to test the regulation of a cell cycle transition: the entry into mitosis (metaphase) [13] (Figure 4). The idea was that a factor called the Maturation Promoting Factor (MPF) was present in the cytoplasm of metaphase-arrested eggs and was produced or activated in a progesterone-dependent manner in prophase-arrested oocytes. This MPF controlled the transition from prophase to metaphase (i.e., interphase to mitosis). Thus, purification of MPF became feasible, allowing new projects to be undertaken to shed further light on how this transpires.

The conclusion of Masui’s team regarding a factor that drives cells to go through the next transition of cell cycle progression was also confirmed by cell fusion experiments performed by Rao and Johnson in 1970. In two manuscripts published in *Nature* [14,15], they reported that fusion of a G1 phase cell and an S phase cell leads to an S phase cell, while fusion of an S phase cell and a G2 phase cell leads to a G2/M phase cell. Even more spectacular, they demonstrated that the cytoplasm of an M phase cell contains a factor that can cause a cell to enter a premature mitosis even when the cell was only in G1. This led to the notion of a cell cycle engine. MPF could now stand for Mitosis Promoting Factor.

The next major step forward again came from Masui’s lab when Manfred Lohka developed an in vitro acellular system that recapitulates cell cycle progression in egg extracts. This opened a plethora of MPF purification opportunities for biochemists [16]. Lohka then moved to James Maller’s lab, where he managed to purify MPF [17]. We will come back to this purification later on.

While biochemists were focusing on MPF purification, geneticists were also studying cell cycle progression, using unicellular organisms such as the budding yeast *S. cerevisiae* and the fission yeast *S. pombe*. Lee Hartwell was working with budding yeast, which gets its name because its cell division starts with the formation of a bud that grows until it reaches the size of the initial cell, then enters mitosis and separates to produce another cell. The size of the bud is directly linked to the progression of the cell cycle: cells with no bud are in G1, cells with a small bud are in S phase, and cells with a large bud are in G2. Lee Hartwell’s plan was to isolate cell cycle thermosensitive mutants generated by mutagenesis, which he called Cell Division Cycle “*cdc*” mutants [18,19,20]. The *cdc28* mutant, for example, attracted his attention because the mutant cells did not divide and were arrested as unbudded cells in G1 before entering S phase.

At the same time, Paul Nurse was working with fission yeast, which gets its name because its cell division looks like bacterial cell division. *S. pombe* cells are rod-shaped cells that elongate during cell cycle progression while maintaining the same diameter, and they eventually divide in the middle when they reach the size of two cells. The length of the cell is hence directly linked to the progression of the cell cycle: small cells are in G1 and long cells are in G2. Just like Lee Hartwell, Paul Nurse identified several genes essential for cell cycle progression, which he also called Cell Division Cycle “*cdc*” genes [21,22].

Paul Nurse identified an interesting mutant, which he called “*wee*” in reference to “small” in Scottish, as he was working in Edinburgh at the time [23]. *Wee1* mutant cells are smaller than the wild-type cells because they enter mitosis prematurely, as if the *wee1* gene product function was to slow down the G2/M transition. One of the *wee* mutants also turned out to be a dominant mutation of cdc2, a previously identified *cdc* mutant [24]. The *cdc2* gene product was then considered to be the key gene for the G2/M transition. With too much Cdc2 activity, cells enter mitosis too early; without Cdc2 activity, cells do not proceed to mitosis. The *cdc2* gene was isolated from an *S. pombe* wild-type cDNA library after rescue of the *cdc2* mutant function, and it was then sequenced [25,26].

Importantly, Paul Nurse reported that *cdc2* was also required for the G1/S transition. Could a single gene control two different transitions of the cell cycle? Could it be the engine of the cell cycle? Is it present in the other yeast, *S. cerevisiae*? Paul Nurse’s team then set out to rescue the *cdc2* phenotype by supplementing the *S. pombe cdc2* mutant with an *S. cerevisiae* gene library. Fortunately, a budding yeast gene was identified and found to be *CDC28.* Not only did these data strongly suggest the universality of the cell cycle controls, they also suggested that *cdc2/CDC28* could be the cell cycle engine that drives cells through cell cycle transitions [25].

**Figure 5:**
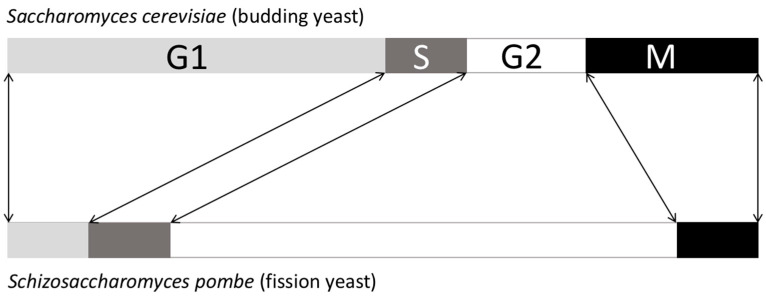
Differences in duration between *S. cerevisiae* and *S. pombe* cell cycle phases.

Then, if Cdc2 is Cdc28 and fulfils the same function, why was it identified as a G1/S actor in budding yeast and a G2/M actor in fission yeast? This answer is found in the difference in the duration of the cell cycle stages between each yeast. Budding yeast has a long G1 and a short G2, while fission yeast has a short G1 and a long G2 (Figure 5).

Statistically, if one works on a factor that controls both the G1/S and the G2/M transitions, it will be identified as a G1/S factor in budding yeast and a G2/M factor in fission yeast because there will be more G1 cells in *S. cerevisiae* cell cultures and more G2 cells in the case of *S. pombe*. Indeed, a rule regarding the cell cycle is that in an asynchronous population of cells, the duration of a cell cycle stage is directly linked to the number of cells in this particular stage. For example, for a cell with a whole cycle duration of 24 h, if in a population of 100 cells (without any G0 cells) one finds 4 cells in mitosis, this will mean that mitosis lasts approximately 1 h (24 h × 4/100 = 0.96 h).

After discovering that the *cdc2/CDC28* gene product is essential for the G2/M transition in both fission and budding yeasts, the next question regards how it exerts its activity.

First of all, what is the *cdc2/CDC28* gene product? Its sequence corresponds to a protein kinase, meaning that, at least in yeast, cell cycle transitions are controlled by protein phosphorylation [27]. This mechanism is conserved in yeasts, but does it extend to all eukaryotic cells? Paul Nurse’s lab reported that human cells also possess a cdc2-like gene that could functionally complement the *cdc2* mutant [28]. It has since even been reported that a plant Cdc2-like kinase is able to complement the *S. pombe* mutant [29]. This unambiguously demonstrates that control of cell cycle progression is highly conserved throughout eukaryotes and that the cell cycle engine is a protein kinase.

## 3. Functional Issues at the Molecular Level

We now return to the biochemists who were purifying MPF from frog egg extracts. Jim Maller, who trained to work on protein kinases with Edwin Krebs, and who established his laboratory in Denver, Colorado, in 1978, was joined by Manfred Lohka from Yoshio Masui’s laboratory. Together, they figured out how to use *Xenopus laevis* egg extract to recapitulate mitosis and to purify MPF [17]. The purification of MPF was achieved in 1988, with the most purified fraction containing two proteins: one of size 34 kDa and the other of size 45 kDa [30]. Many different laboratories around the world joined the race to purify and identify MPF, and the first MPF protein to be identified was the 34 kDa protein purified from *Xenopus laevi*, which turned out to be a homolog of the yeast cdc2 kinase. This was first demonstrated by using the *S. pombe p13suc1* gene product, known to interact with Cdc2, as a bait to search for binding partners in *Xenopus* egg extract. *Xenopus* Cdc2 was then identified, but it was again associated with a 42 kDa unknown protein partner [31]. Another approach was to use an antibody raised against *S. pombe* Cdc2 and test its cross-reaction against the purified *Xenopus* MPF (34 and 45 kDa proteins), and again the antibody reacted with the 34 kDa protein, thus identifying it as the *Xenopus laevis* Cdc2 [32].

The question then was: what is the other protein?

Meanwhile, Tim Hunt, who was interested in protein synthesis, started to analyse the process in sea urchin eggs undergoing cleavage after fecundation. In 1982, he was surprised to observe proteins that were newly synthesised before each division but, most importantly, that were immediately and totally degraded after each cleavage, and then resynthesised again, etc. Because of its cycling appearance, he called this protein ‘cyclin’ [33]. He knew he had discovered something important for the cell cycle field but he was at a bit of a loss as to how it was working (from Woods Hole Lab, Tim Hunt’s personal letter to his friend Richard Jackson). The relationship between MPF and protein degradation was then first suggested by the team of Marcel Dorée in 1985: “… proteases might be involved in… the drop in MPF activity…”, he suggested [34]. Then it took time to understand the mechanism underlying the activation and inactivation of MPF activity, until several labs discovered that a cyclin protein associates with cdc2 kinase to form MPF, and that the 45 kDa protein of Xenopus MPF was the same as cyclin B [35,36,37,38].

**Figure 6:**
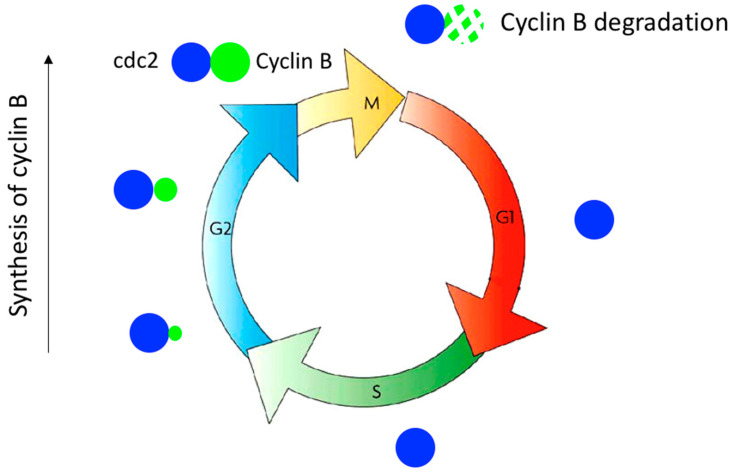
Control of the G2/M transition by cdc2/cyclin B. Cyclin B is progressively synthesised during G2 and binds to cdc2 to activate its kinase activity, thereby triggering entry into mitosis. Cyclin B is then rapidly degraded, leading to inactivation of cdc2 kinase, which triggers exit from mitosis.

The engine for cell cycle progression had therefore been discovered: a protein kinase that must associate with a cyclin to become catalytically active. The protein kinase is present throughout the cell cycle, and when the cyclin is synthesised, it binds to the kinase and activates it, allowing the kinase to perform its function by controlling a cell cycle transition; the kinase is then inactivated by degradation of the cyclin when the transition has taken place (Figure 6). This mechanism that controls cell cycle transition was discovered while studying the entry into mitosis. Cdc2-cyclin B controls the G2/M transition, with cyclin B being synthesised during G2 and degraded when the cell exits mitosis [39].

In the late 1980s, many labs were screening libraries to identify cdc2 in different species. It should be pointed out that DNA sequencing was still being performed in the lab at that time, but its efficiency was greatly and rapidly improving. During these searches for cdc2, many labs were disappointed at first as they were indeed identifying cdc2-related kinases, but these could not rescue yeast cdc2 mutants [40]. However, obvious questions began to arise: how do cells differentially control the two major G1/S and G2/M transitions with a single kinase? This problem was first solved in yeast, where the transitions are controlled by the single cdc2 kinase associated with separate cyclins for G1/S and G2/M. Thus, two different cyclins are involved to construct two different cdc2/cyclin kinases. The situation is different in higher eukaryotes, for which the genomes were found to encode several cdc2-like kinases, leading to the idea that the cell cycle transitions in multicellular eukaryotes are controlled by different cdc2-like kinases [40,41]. Progress came again from yeast when it was observed that a cdc2-related kinase could partially complement the cdc28 mutant in *S. cerevisiae* (the *S. pombe* homologue of cdc2) [42,43]. It was also observed that mouse cells with a thermosensitive mutation of cdc2 arrested only in G2 phase at the restricted temperature and not in G1 phase. This strongly suggested that another endogenous cdc2-like entity controlled the G1/S transition [44]. The cdc2-related kinases in higher eukaryotes were then all called cdks for cyclin-dependent kinases, and since cdc2 was discovered first, it was called cdk1. The second cdk, the homolog of Eg1 in *Xenopus laevis*, was called cdk2 [40].

**Figure 7:**
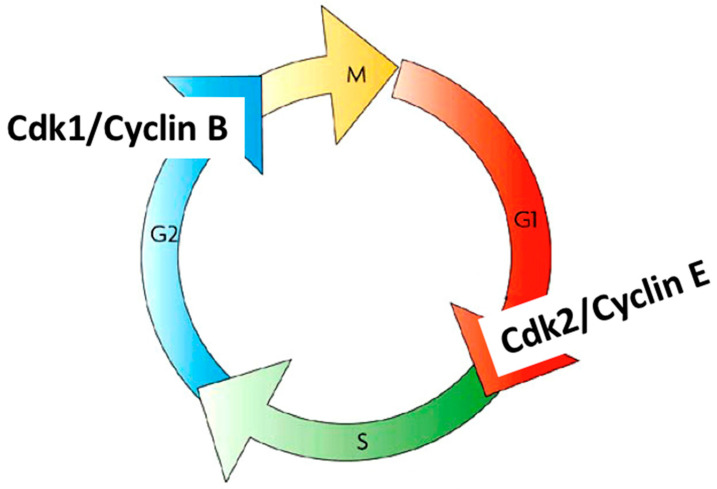
Control of the G1/S transition by cdk2/cyclinE and the G2/M transition by cdk1/cyclin B.

In the early 1990s, it became evident that cdk1 and cdk2 associate with different cyclins to control different cell cycle transitions in human cells. It was demonstrated, for instance, that cdk2/cyclin E controls the G1/S transition and cdk1/cyclin B controls the G2/M transition [45,46] (Figure 7). The understanding of how the cell cycle progresses then accelerated. To date, approximately 20 different cdks and cdk-like entities and approximately 30 different cyclins and cyclin-like proteins have been identified [47,48]. Although not all cdks can associate with all cyclins, the number of possible cdk/cyclin combinations is very high, thus revealing the complexity of cell cycle regulation by these kinases. Not only is the regulation complex, but these kinases are redundant and can sometimes replace each other. For a long time, it was thought that cdk1 was the original and only kinase driving the cell cycle in higher eukaryotes [49], but a recent publication from Randy Poon’s lab has shown that cdk2 can replace cdk1 [50].

## 4. Checkpoints

It soon became clear that cell cycle progression is not controlled by a single engine but by several, and that each one controlled only a small part of cell cycle progression. At each transition in the cycle, an engine is assembled and then disassembled once the transition is complete. To undergo the next transition, the cell assembles a new engine, and so on.

At the molecular level, these engines correspond to protein kinases in the form of cdk/cyclin complexes. These complexes act as follows: a cyclin is synthesised and then binds to a cdk, thereby generating an active cdk/cyclin kinase complex that phosphorylates substrates, which, in turn, triggers a first cell cycle transition. Once the transition has taken place, the cyclin is degraded, thereby leading to inactivation of the kinase. Then, a second cyclin is synthesised and forms another active cdk/cyclin kinase complex that phosphorylates other substrates, which then trigger a second transition. Once this is completed, the cyclin is degraded, leading to inactivation of the kinase, and so on. Each cdk/cyclin pair has different substrates, which leads to the activation of different signalling pathways that control different transitions.

The question quickly arose as to whether this mechanism was sufficient to explain the reproducibility of the G1-S-G2-M sequence of cell cycle progression and the robustness of the overall mechanism. Also, what happens if something goes wrong during the cell cycle progression?

To solve these problems, Lee Hartwell introduced a novel concept in the mid-1970s: the dependence between events occurring during the progression of the cell cycle, e.g., a cell cannot enter M phase if S phase has not been fully completed. The introduction of a completion condition in the regulation of cell cycle progression was a breakthrough and logical given the reproducibility of the mechanisms and the conserved order of events that ultimately lead to cell division [20,51].

How would this work?

The idea is that cells have monitoring mechanisms that continuously control the progression of biological events, as well as their completion, during cell cycle progression. Lee Hartwell called these mechanisms “checkpoints” that must be always active and distributed throughout the progression of the cell cycle; only the completion of an event can deactivate the corresponding checkpoint, thereby allowing the cell cycle to continue its progression [50].

As Lee Hartwell further pointed out, a checkpoint cannot be detected simply by observing cells progressing through a normal cell cycle. Only by disrupting an event that occurs during progression can the presence of a checkpoint be detected. For example, inhibition of an essential cell cycle mechanism induces a slowing of progression or a cell cycle arrest. The cell then waits for the inhibition to disappear before resuming its progression.

Checkpoints are quality control mechanisms that detect errors generated during cell cycle progression and intervene in the engines of the cycle to slow or stop them, thereby giving the cell time to correct these errors.

In the presence of checkpoints, cells have only two options for progression through the cell cycle: satisfy the checkpoint or remove the checkpoint. This concept immediately implies that checkpoints are not essential. Indeed, in a perfect world, checkpoints should not be necessary, their function being to detect defects and adapt the cell cycle progression accordingly [52].

There are two main rules to consider when looking at a cell cycle arrest that may be due to a checkpoint.

The first rule is that a cell cycle arrest due to a checkpoint can be overridden. For example, the addition of a drug that inhibits an enzyme involved in a given checkpoint allows cycle progression to resume. This is not the case when the arrest is due to a defect in the cell cycle engine, e.g., a defect in cdk/cyclin cannot be overridden.

The second rule is that checkpoints are always active during the progression of the cell cycle. Thus, whenever the cell encounters a checkpoint, the only way to go through it and continue with cell cycle progression is to satisfy the checkpoint. Many scientists have often misinterpreted the checkpoint concept by stating that a checkpoint can be activated in order to arrest the cell cycle. This is not the case. Introducing defects in the cell does not result in activation of the checkpoint in charge of the defect. Rather, it results in a checkpoint being maintained.

There are four main checkpoints: the G1/S checkpoint, the G2 checkpoint, the mitotic checkpoint, and the abscission checkpoint.

(1) The G1/S checkpoint is also known as the restriction point (or START in yeast). In the early 1970s, Howard Temin observed that adding serum to resting chicken cell cultures induced a G1/S transition [53]. Three years later, Arthur Pardee defined this restriction point as the only place in the cycle where the cell can go from a proliferating state to a resting state and vice versa [54]. For instance, removing serum from proliferating cells induces a G1 arrest at the restriction point, while adding serum to resting cells induces a G1/S transition and a commitment to proliferate. This checkpoint monitors whether there are enough nutrients and enough growth factors for the cell to enter the cell cycle and divide. All cells that have already committed to dividing will continue their progression in the cycle but will then arrest at the next G1.

(2) The G2 checkpoint is also known as the DNA damage checkpoint. This checkpoint monitors the presence of lesions in the DNA, and it only authorises entry into mitosis to cells that have undamaged DNA. In 1989, to identify genes involved in this checkpoint, Lee Hartwell used *S. cerevisiae* cells in which he induced DNA damage by X-ray exposure. Under these conditions, the cell cycle progression arrested in G2 phase. In order to identify genes involved in this G2 arrest, he designed a screen to look for temperature-sensitive yeast mutants that failed to arrest cell cycle progression at the restrictive temperature and, hence, kept dividing in the presence of DNA damage (i.e., they were unable to detect the defect). He identified *RAD9* as one of the genes essential to this arrest in G2 [55,56]. This finding was an important breakthrough for the cell cycle field because it revealed that cells have developed quality control mechanisms to check cell cycle progression that ensure its reproducibility.

(3) The mitotic checkpoint is also known as the spindle assembly checkpoint. Again in the 1970s, Raymond Zirkle observed that UV destruction of the spindle caused a delay in anaphase that depended on the timing of the irradiation, i.e., before or after attachment of the last kinetochore to spindle microtubules [57]. This checkpoint monitors the attachment of chromosome kinetochores to microtubules, and it is only satisfied when all kinetochores are attached; only when this condition is satisfied does the cell progress from metaphase to anaphase. Conly Rieder’s work has long been dedicated to the definition of this checkpoint [58,59,60].

(4) The cytokinesis checkpoint is also known as the NoCut checkpoint. Discovered in *S. cerevisiae* by Yves Barral in 2006, this checkpoint monitors the presence of chromosome bridges at the cleavage plane to avoid chromosome breakage during abscission [61,62]. The main player in this checkpoint is Aurora-B kinase, which is inactivated when the checkpoint is satisfied [63,64].

## 5. Conclusions

**Figure 8:**
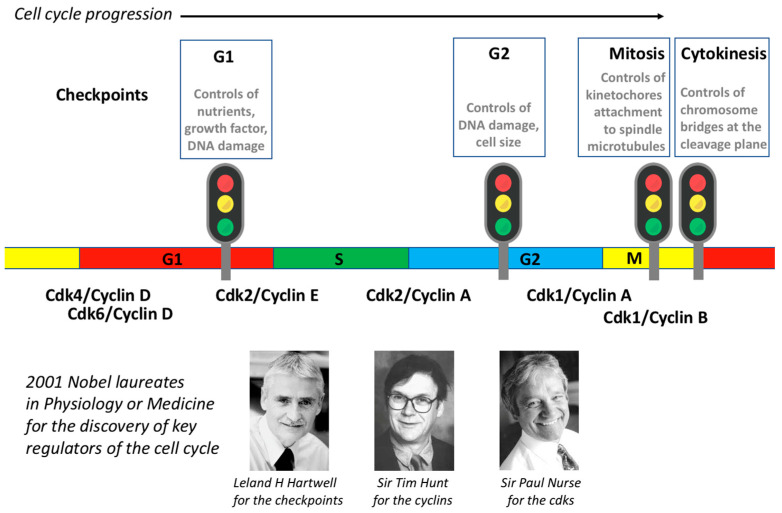
Summary of the major cell cycle controls: cdks, cyclins, and checkpoints and the Nobel laureates for these cell cycle discoveries.

The cell cycle comprises a series of coordinated events that result in the division of an original (mother) cell into two newly generated (daughter) cells. It is a mechanism universally shared by all eukaryotic cells. Progression of the cell cycle is driven by several cdk/cyclin complexes that take turns to ensure transitions from one step to the next. The robustness and reproducibility of the cell cycle are ensured by quality control mechanisms called checkpoints, which only when satisfied allow the cell cycle to progress. In 2001, Prof. Lee Hartwell, Sir Tim Hunt, and Sir Paul Nurse were awarded the Nobel Prize in Physiology or Medicine for their contributions to the discovery of key regulators of the cell cycle (Figure 8).
